# Tightly Constrained Genome Reduction and Relaxation of Purifying Selection during Secondary Plastid Endosymbiosis

**DOI:** 10.1093/molbev/msab295

**Published:** 2021-10-06

**Authors:** Kavitha Uthanumallian, Cintia Iha, Sonja I Repetti, Cheong Xin Chan, Debashish Bhattacharya, Sebastian Duchene, Heroen Verbruggen

**Affiliations:** 1 School of BioSciences, University of Melbourne, Melbourne, VIC, Australia; 2 Australian Centre for Ecogenomics, School of Chemistry and Molecular Biosciences, The University of Queensland, Brisbane, QLD, Australia; 3 Department of Biochemistry and Microbiology, Rutgers University, New Brunswick, NJ, USA; 4 Deptartment of Microbiology and Immunology, Peter Doherty Institute for Infection and Immunity, University of Melbourne, Melbourne, VIC, Australia

**Keywords:** secondary endosymbiosis, plastids, photosynthetic organelle, selection efficiency variation, genetic drift

## Abstract

Endosymbiosis, the establishment of a former free-living prokaryotic or eukaryotic cell as an organelle inside a host cell, can dramatically alter the genomic architecture of the endosymbiont. Plastids or chloroplasts, the light-harvesting organelle of photosynthetic eukaryotes, are excellent models to study this phenomenon because plastid origin has occurred multiple times in evolution. Here, we investigate the genomic signature of molecular processes acting through secondary plastid endosymbiosis—the origination of a new plastid from a free-living eukaryotic alga. We used phylogenetic comparative methods to study gene loss and changes in selective regimes on plastid genomes, focusing on green algae that have given rise to three independent lineages with secondary plastids (euglenophytes, chlorarachniophytes, and *Lepidodinium*). Our results show an overall increase in gene loss associated with secondary endosymbiosis, but this loss is tightly constrained by the retention of genes essential for plastid function. The data show that secondary plastids have experienced temporary relaxation of purifying selection during secondary endosymbiosis. However, this process is tightly constrained, with selection relaxed only relative to the background in primary plastids. Purifying selection remains strong in absolute terms even during the endosymbiosis events. Selection intensity rebounds to pre-endosymbiosis levels following endosymbiosis events, demonstrating the changes in selection efficiency during different origin phases of secondary plastids. Independent endosymbiosis events in the euglenophytes, chlorarachniophytes, and *Lepidodinium* differ in their degree of relaxation of selection, highlighting the different evolutionary contexts of these events. This study reveals the selection–drift interplay during secondary endosymbiosis and evolutionary parallels during organellogenesis.

## Introduction

The endosymbiosis event leading to present-day chloroplasts is inferred to have taken place ∼1.5 billion years ago through the incorporation of a cyanobacterium by a heterotrophic host (Yoon et al. 2004; Price et al. 2012; Nowack and Weber 2018). This endosymbiosis event is referred to as primary endosymbiosis, with the plastids of the organisms descending from this event termed primary plastids (Archibald 2009; Keeling 2010). Three photosynthetic lineages emerged from this ancestor: the Chlorophyta (green algae), Rhodophyta (red algae), and Glaucocystophyta. Subsequently, several red and green algae have undergone secondary endosymbiosis events, giving rise to complex plastids. Secondary endosymbiosis differs in having a eukaryotic alga (carrying a primary plastid) as the photosynthetic partner being established as an organelle, and this process has spread photosynthesis to many unrelated branches of the eukaryotic tree of life (Keeling 2010). Despite the relevance of plastid endosymbiosis for eukaryotic evolution and algal diversity, understanding of molecular evolution during the origination of these plastids is limited.

Endosymbionts often experience lowered levels of natural selection (Latorre and Manzano-Marín 2017; Wernegreen 2017), with the elevation of levels of stochastic genetic drift leading to an accumulation of slightly deleterious mutations, resulting in genome reduction and making them more susceptible to degradation (Moran 1996; Pettersson and Berg 2007; Moran et al. 2008; Bennett and Moran 2015). Plastids have retained a highly reduced genome (ca. 100–200 kb) characterized by accelerated rates of evolution and AT-biased nucleotide composition compared with free-living cyanobacteria (Green 2011; Bennett and Moran 2015). As is the case in many endosymbionts, plastid genomes have lost the majority of cyanobacterial genes, some having been transferred to the nucleus. Some of the gene losses are compensated by nucleus-encoded plastid-targeted proteins that enable the integration of plastids into the host cell biology. Plastid genomes encode a highly conserved set of key genes encoding for core photosystem components, ATP synthesis, and protein translation (Allen 1993, 2017), which are under strong purifying selection (Smith 2015; Grisdale et al. 2019). It has been hypothesized that the retention of genes in the plastid genome enhances the ability of organelles to efficiently respond to fluctuating conditions (Allen 1993, 2017; Johnston 2019). Strong purifying selection on retained plastid genomes distinguishes them from most other endosymbiont genomes in early stages of endosymbiosis. Although parallels can be expected between the evolutionary forces acting during the establishment of plastid endosymbiosis (Reyes-Prieto et al. 2010; Lhee et al. 2019) and other obligate endosymbiosis events based on the similarities in their overall genomic features, there has been very little work on characterizing patterns of selection and drift in the origination of plastid organelles.

Secondary endosymbiosis differs fundamentally from primary because, at the start of this process, the genomes of the primary plastid have already transitioned to a reduced state (Green 2011), with secondary green plastids having a roughly similar gene content to primary green plastids (Suzuki et al. 2016; Karnkowska et al. 2018). Inouye and Okamoto postulate that secondary endosymbiosis of plastids consists of several stages, including permanent retention of the engulfed primary alga, followed by reduction of the endosymbiont genomes (primarily the nucleus) and ultimately fixation as an organelle through nuclear-encoded plastid-targeted genes (Inouye and Okamoto 2005). Recent studies have emphasized the possible role of the secondary host nucleus in facilitating the integration of the incoming green plastids in lineages that have hosted other plastids before (Ponce-Toledo et al. 2018, 2019). All these previous studies related to secondary endosymbiosis are focused on the reduction of the endosymbiont’s nuclear genome, but the molecular evolution of plastid genomes through the various stages of secondary endosymbiosis remains largely unexplored.

This study aims to characterize the molecular evolutionary processes acting on the origin of secondary plastids, using secondary plastids of green algal ancestry as a model system. The secondary plastids of green algal ancestry are present in three lineages, the chlorarachniophytes (a group of Rhizaria), the euglenophytes (a group of Discoba), and the dinoflagellate genus *Lepidodinium* (Jackson et al. 2018). The existence of these three independent evolutionary events, with clearly identifiable host and plastid donor origins, makes green-type secondary plastids an excellent case study to investigate shared and divergent features associated with secondary endosymbiosis events. Here, we use phylogenetic methods to examine the variation in selection on genes before, during and after endosymbiosis, and to compare how this selection varies across genes and endosymbiosis events. We also quantify patterns and rates of gene loss across these events of secondary endosymbiosis. Our results are interpreted in the light of evolutionary processes that can contribute to variation in selection during secondary endosymbiosis.

## Results and Discussion

### Plastid Genome Features

Most plastid genomes, including those of secondary plastids, are small in size (median 153 kb), have low GC content (median 0.34), and they encode an average of 80 identified protein-coding genes. Plastid genomes of chlorarachniophytes (median 70 kb genome, median 60 CDS) and *Lepidodinium* (66 kb, 62 CDS) are smaller with fewer CDS than in euglenophytes plastid genomes (median 90 kb, median 64 CDS) (supplementary table S1). Codon usage bias estimated using synonymous codon usage showed that all green plastids studied had similar codon usage bias, which appeared proportional to nucleotide composition (supplementary fig. S1). Among the secondary plastid lineages, chlorarachniophyte plastids had slightly lower GC content and higher codon usage bias than euglenophytes and *Lepidodinium*. However, codon usage bias for secondary plastids was within the range of that observed for primary plastids.

### Tightly Constrained Genome Reduction

**Fig. 1.**
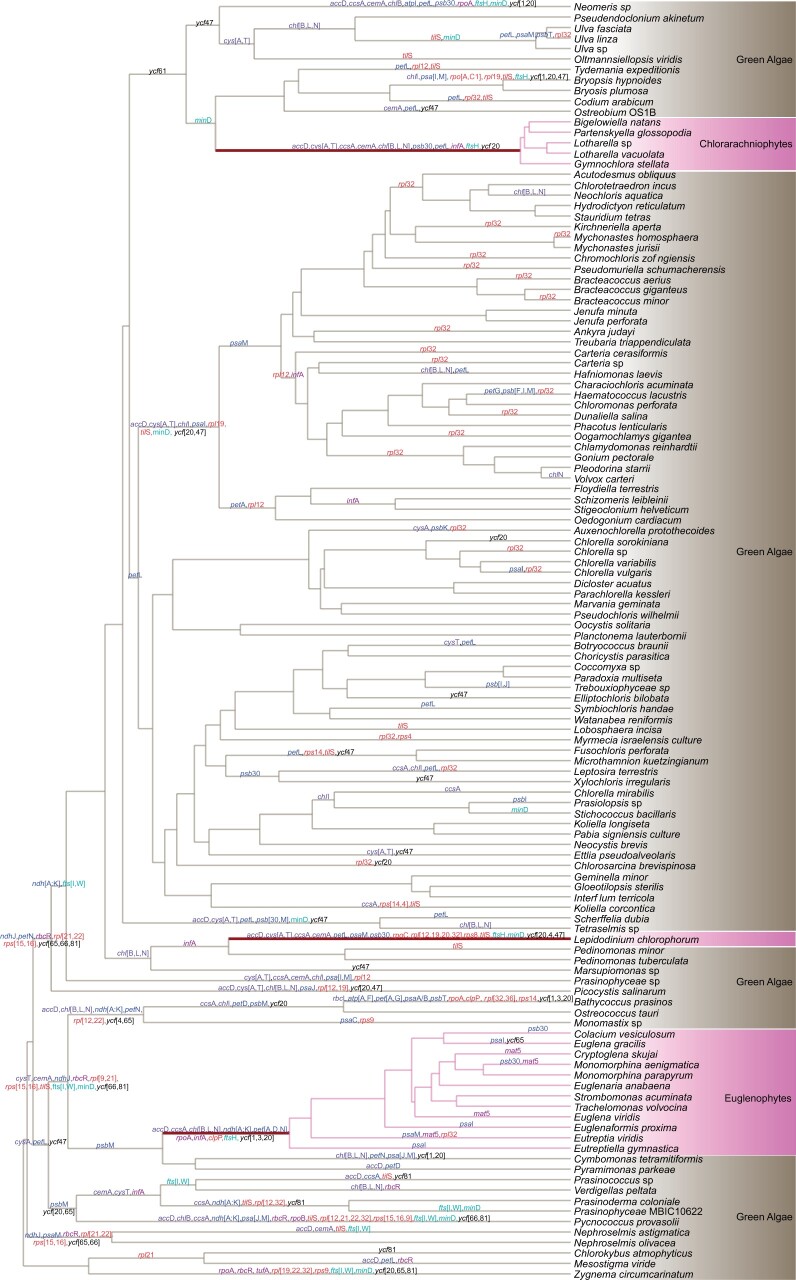
Evolution of green-type plastids across secondary endosymbiosis events. The phylogeny includes lineages that have primary plastids (i.e., green algae, in wheat brown), branches with secondary plastids (i.e., Chlorarachniophytes, *Lepidodinium*, Euglenophytes, in pink), and branches along which endosymbiosis happens (maroon). Inferred losses of named genes are indicated. Losses of unnamed conserved open reading frames are not listed here but are included in the gene loss counts presented in the text. The color scheme used for the gene losses are—photosystems (blue), cell and organelle division (aqua), genetic system (purple), ribosomal (red), metabolism and transport (violet), and others (black).

**Fig. 2.**
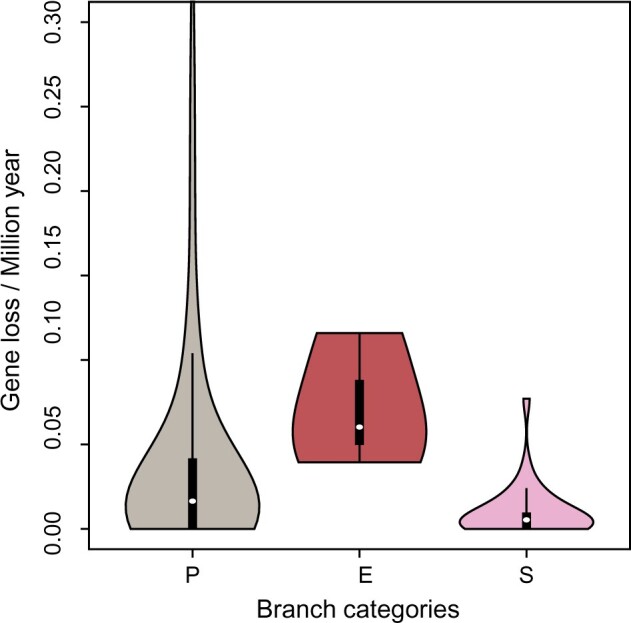
Inferred rates of gene loss in branches with primary plastids (P), secondary plastids (S), and branches along which endosymbiosis (E) takes place.

By clustering the translated proteins into homologous groups and estimating gene loss with Dollo parsimony, it became apparent that plastid genomes underwent an elevated level of genome reduction during secondary endosymbiosis events, but that they retained all key plastid genes encoding for core subunits related to photosynthesis, ATP, and protein synthesis (figs. 1 and 2). Reductive genome evolution highlights the similarities in molecular evolution between secondary plastid endosymbiosis and many examples of bacterial endosymbiosis in insects (McCutcheon and Moran 2011). Gene loss is particularly severe during primary endosymbiosis, with cyanobacterial genomes (ca. 1,800–12,000 genes) reduced to about 80–230 genes found in primary plastids (Gabr et al. 2020). Gene loss from plastids along the endosymbiotic branches representing the evolutionary period in which the organelle gets integrated into a new host (maroon branches in fig. 1), was small in comparison. Our estimates indicate that chlorarachniophytes lost 29 genes during secondary endosymbiosis followed by euglenophytes with 26 gene losses and *Lepidodinum* with 22 (fig. 1). Even though the endosymbiotic branches are among the top five branches losing the most genes, the difference compared with the background is not statistically significant (ANOVA and Tukey HSD tests), possibly due to the small sample size (*n* = 3) of endosymbiotic branches available for analysis.

When viewed as the rate of gene loss per million years, the endosymbiosis branches had somewhat higher rates on average (fig. 2), but ranked lists showed that chlorarachniophytes and *Lepidodinium* were not among the branches with the fastest rates of loss. Therefore, despite most gene losses occurring on the endosymbiotic branches, the rates of loss per million years for these branches are not particularly high, suggesting that gene loss is a punctuated process occurring early in endosymbiosis (Moran and Mira 2001; Oakeson et al. 2014). When correcting for the branch lengths of endosymbiotic branches, this punctuated effect is diluted to the point of not differing from background rates. Interestingly, the three independent endosymbiosis events showed similar numbers of gene losses (between 22 and 29), adding to a list of similarities between secondary endosymbiosis events that also includes the convergent evolution of nucleomorph architecture seen in chlorarachniophytes and cryptophytes (Sarai et al. 2020; Sibbald and Archibald 2020).

Our gene loss analysis showed that 17 genes were lost more than ten times across the phylogeny, including *rpl*32 (ribosomal protein, 30 times), *til*S (tRNA Ile-lysidine synthetase, 18), *pet*L (cytochrome b6-f complex, 16), and *ycf*47 (14). Only *accD* (lipid acid synthesis), *ccsA* (mediates heme attachment to c-type cytochromes), and *ftsH* (cell division) were lost in all three endosymbiotic events. Some genes lost during one endosymbiotic event are also absent from other secondary plastids but were lost before the endosymbiotic event. For instance, *ndh* (NAD(P)H oxidoreductase) was lost during euglenophyte endosymbiosis but was also lost from the green algal lineages that gave rise to the chlorarachniophytes and *Lepidodinium*. Most of the genes lost during secondary endosymbiosis are likely to be compensated by nuclear homologs or through an alternative pathway. For instance, the light-independent chlorophyll synthesis genes *chl*B, *chl*L, and *chl*N that were lost during chlorarachniophyte and euglenophyte endosymbiosis and in many other primary plastids, including the ancestors of *Lepidodinium*, can be compensated by the light-dependent chlorophyll production pathway (Hunsperger et al. 2015). The *chl*B, *chl*L, and *chl*N genes have also been lost from some secondary plastids of cryptophyte algae (Fong and Archibald 2008).

Homologs of *rpl*12, *rpl*32, *rps*9 (small ribosomal proteins), *inf*A (translational initiation factor), and *fts*H were found in the nuclear genome of the chlorarachniophyte *Bigelowiella natans* (Curtis et al. 2012), suggesting they may have been transferred from the plastid rather than lost entirely. A previous study recovered the homologs of *pet*A, *pet*N, *ycf*3, *clp*P (Clp protease proteolytic subunit), and *fts*H in the transcriptomes of the euglenophytes *Euglena gracilis* and *Eutreptiella*, suggesting that these genes were transferred to the nucleus (Hrdá et al. 2012) and scanning of the *E. gracilis* genome corroborated these results (Novák Vanclová et al. 2020). Aside from the genes mentioned above, all other genes lost during secondary endosymbiosis including those with a function in photosynthesis (e.g., *psb*30, *psb*M, and *psa*I) were not detected in the nuclear genomes of *B. natans* and *E. gracilis*, and may represent genuine gene losses, but some caution is warranted because most of these proteins are small and may be missed in genome-wide searches using BLAST.

Several of the genes predicted to be lost during secondary endosymbiosis (*acc*D, *inf*A, *ndh*, *ycf*1, *ycf*3, and *ycf*4) were also lost from plastid genomes in other lineages, and compensatory nuclear-encoded genes have been identified (Boudreau et al. 1997; Millen et al. 2001; Martín and Sabater 2010; Huerlimann and Heimann 2013). Our gene loss analyses confirm previous work showing that gene loss from plastids is common. The nuclear-encoded plastid-targeted proteins compensating for these losses are diverse in nature and vary between lineages and among genes. Large contributions of noncyanobacterial proteins to the plastid proteome of algae with primary plastids (*Paulinella* and Archaeplastida) highlight the importance of HGT from other bacteria during primary endosymbiosis (Qiu et al. 2013; Nowack et al. 2016). Studies of *E. gracilis* (Novák Vanclová et al. 2020) and *B. natans* (Curtis et al. 2012) support involvement of HGT from partners other than the plastid donor in secondary plastid endosymbiosis and highlight the role of host-derived genes that are repurposed or duplicated during endosymbiont recruitment. The shopping-bag and allied hypotheses support the possible role of genes that have accumulated in the host nucleus, either by EGT or HGT during previous transient or cryptic endosymbiosis events, in compensating gene losses (Larkum et al. 2007; Ponce-Toledo et al. 2018, 2019). Previous studies indicate the existence of a red endosymbiont in the ancestors of euglenophytes, chlorarachniophytes, and *Lepidodinium* (Kamikawa et al. 2015; Ponce-Toledo et al. 2018, 2019) but the extent to which processes related to the shopping bag hypothesis have impacted upon these independent events requires further investigation. All these findings support the notion that the host plays a major role during early stages of plastid endosymbiosis (Tyra et al. 2007; Gross and Bhattacharya 2009). Once a more diverse set of nuclear genomes for the host and green algal lineages involved in these secondary endosymbiosis events becomes available, nuclear genome dynamics through secondary endosymbiosis can be investigated in more detail and the relative contribution of these different strategies for compensating plastid gene losses clarified.

Losses of genes for which functions can be compensated are likely to have little impact on plastid function. Loss of similar genes in parallel in different parts of the tree suggests they may experience reduced selective constraints compared with key photosynthesis genes, and in periods with increased drift, such genes may be more likely to be lost than genes under stronger selection. Recent work shows that genes encoding central subunits of the electron transport chain are more likely to be retained in the organelle (Johnston and Williams 2016). In line with this result, we observed that across the green algal phylogeny, 48 genes including the core components of photosynthesis, and protein synthesis are highly conserved (never lost or lost once). Loss of photosynthetic genes from plastid genomes happens occasionally in green plastid lineages, but this is as part of a transition to a nonphotosynthetic lifestyle from photosynthetic ancestors (Sibbald and Archibald 2020), and not in direct association with the secondary endosymbiosis events studied here. Retention of a plastid genome in *Euglena longa*, a nonphotosynthetic euglenophyte that has lost photosynthesis-related genes, hints at the selective pressures on plastid genomes beyond photosynthesis and reflects the underlying lifestyle alterations to secondary heterotrophy (Zahonova et al. 2018; Fussy et al. 2020).

The role of selection in retaining genes has also been demonstrated in the chromatophore genomes of *Paulinella*, a model species for the study of primary endosymbiosis (Reyes-Prieto et al. 2010; Valadez-Cano et al. 2017; Lhee et al. 2019). Overall, our results suggest that genome reduction appears to be elevated during secondary endosymbiosis but is a tightly constrained process with strong selection to retain genes with key functions. Of course, the lineages with secondary endosymbionts that we study here are all photosynthetic, implying that by the design of our study, we introduced a bias towards endosymbiosis events that would have maintained all genes with an essential function in photosynthesis. It is perfectly conceivable that other outcomes are possible in endosymbiosis events that involve loss of photosynthetic function, but we are not aware of any instances where a cyanobacteria or eukaryotic alga has been retained as an endosymbiont for functions other than photosynthesis, besides secondarily nonphotosynthetic groups such as the apicomplexans. *Rhopalodia* species may be an exception because the intracellular spheroid bodies that these diatoms contain represent a vertically transmitted endosymbiont of cyanobacterial origin, which performs nitrogen fixation, not photosynthesis (Prechtl et al. 2004; Nakayama and Inagaki 2017).

### Selection Dynamics through Endosymbiosis

**Fig. 3.**
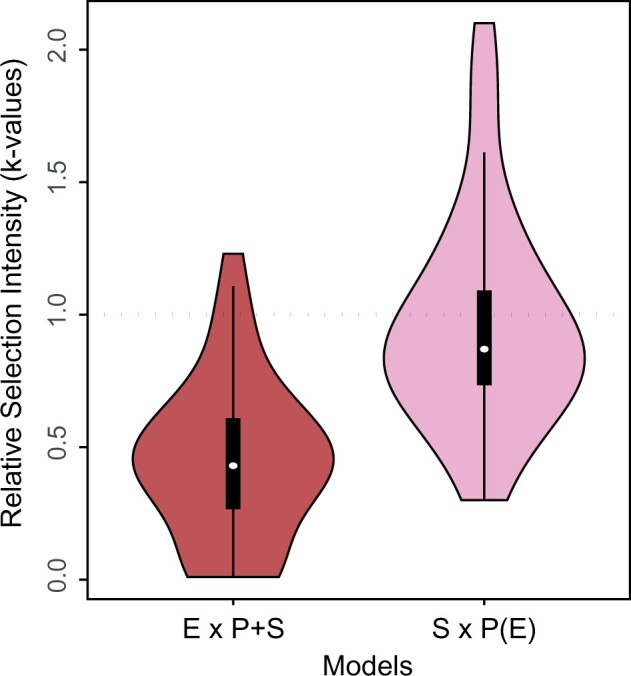
Distribution of the relative selection intensity parameter (*k*) values of the Hyphy RELAX model for 1) Endosymbiosis (test) versus Primary and Secondary branches (reference) [E × P + S model], and 2) Secondary(test) versus Primary (reference), excluding endosymbiosis branches [S × P(E) model]. Selection intensity is relaxed when *k* < 1 or intensified when *k* > 1. These plots show that endosymbiosis branches have relaxed selection compared with the primary and secondary branches and that selection on secondary branches is similar to that of primary branches, indicating that the relation of selection during endosymbiosis is temporary.

For our analysis of selection dynamics through endosymbiosis, the phylogeny was divided into three sets of branches representing primary plastids (P), secondary plastids (S), and endosymbiosis branches (E). Selection intensity during secondary endosymbiosis was quantified using a Hyphy RELAX model that contrasts the selection on the endosymbiosis branches relative to all other branches. The relative selection intensity parameter (*k*-value) of the fitted model showed that the distribution of *k*-values across genes is well below 1 (median 0.43), a clear signature of relaxation of selection in the endosymbiotic branches (E) compared with all other branches (P + S) (fig. 3 and see table 1, supplementary table S2). Of the 34 genes in the analysis, 26 showed statistically supported relaxation. Two outlier genes (*psb*D and *psb*E) showed slight intensification of selection (*k* > 1) for this model, but without significant statistical support. The same model (denoted E × P + S) applied to a concatenated alignment of all plastid genes (supplementary table S3) returned results in line with the findings for individual genes, with relative selection intensity parameter (*k*) value of 0.55. The E × P + S model is a significantly better fit to the concatenated sequences than the null model (*P *<* *0.0001 and likelihood ratio = 557.65), implying a significant decrease in evolutionary selection (relaxation) during endosymbiosis.

**Table 1.** The Number of Green Algal Plastid Genes Showing Relaxation and Intensification of Selection for Each Model.

Model	Relaxation (*k* < 1)	Significant Relaxation	Intensification (*k* > 1)	Significant Intensification	Neither (*k* = 1)
E × P + S	32	26	2	—	—
S × P(E)	19	13	12	9	3

Although the signature of relaxation is clear, this does not imply that molecular evolution is neutral in endosymbiotic branches. The model categorized 82.12% of sites as being under purifying selection, with *ω* (ratio of nonsynonymous (d*N*) to synonymous (d*S*) substitutions) value of 0.06 in the endosymbiotic branches indicating that most sites remain under purifying selection even during endosymbiosis events. BUSTEC analyses provided additional statistical support for purifying selection along endosymbiosis branches, with all genes having lower AIC scores for the unconstrained model with purifying selection than for the model constrained to exclude purifying selection (supplementary table S4).

Selection analysis based on the E × P + S model and the BUSTEC results helps to characterize the molecular evolutionary process during secondary plastid endosymbiosis. Studies of insect endosymbionts suggest that relaxation of purifying selection during endosymbiosis establishment in obligate endosymbionts of insects can be due to two processes: a population bottleneck and decrease in functional constraints on proteins (Moran 1996; Wernegreen 2004, 2015). From the observations of purifying selection and tight constraints on gene loss, we can infer that selection continued to act on plastid genomes during secondary endosymbiosis—likely a reflection of the continued roles of plastids as photosynthetic partners throughout the process. Consequently, it appears unlikely that the relaxation reflects a change in functional constraints on the retained genes. Also, the relaxation of selection is observed on nearly all retained genes, further shifting the balance of evidence towards population size effects on the evolution of plastid genomes during endosymbiosis. The near-neutral theory predicts that in small populations, the fate of near-neutral mutations depends on the balance between selection and the stochastic effect of drift (Ohta 1972, 1992). During bottlenecks, one can expect strongly deleterious mutations to continue being eliminated, whereas slightly deleterious mutations will have higher chances of being fixed in the population by stochastic drift than being eliminated by selection (Woolfit and Bromham 2003). In the chloroplast genes studied here, one would expect this process to result in more nonsynonymous substitutions in the endosymbiotic branches, in line with the reduced selection efficiency we observe.

Relative selection analysis using a different model comparing secondary plastids to primary plastids (denoted S × P[E]) suggests that relaxation of selection during endosymbiosis is temporary, indicated by the distribution of *k*-values that encompasses 1 (median 0.87) and more-similar numbers of genes that were relaxed (13), intensified (9), or inconclusive (9) in secondary branches. The analysis on concatenated sequences showed similar results (median *k* = 0.96) and was not preferred over the null model, providing a clear indication that following the relaxation during endosymbiosis, the purifying selection regime on plastid genes returns to values similar to those before endosymbiosis.

Comparative studies of the genomes of endosymbionts at different stages of integration have shown that genome stability increases with the age of the endosymbiont and suggested that this may be due to selection (Allen et al. 2009; Martínez-Cano et al. 2015). Our findings agree with these observations, and our model system has the added advantage of the endosymbiont becoming a stable organelle, fully integrated, and codiversifying with the host following endosymbiosis, which was not the case in the previously studied endosymbiont models. This allowed us to disentangle the molecular dynamics along the endosymbiosis branch from that of a stable integrated secondary plastid, showing that the purifying selection regime rebounds to near pre-endosymbiosis levels once the organelle is established.

**Fig. 4.**
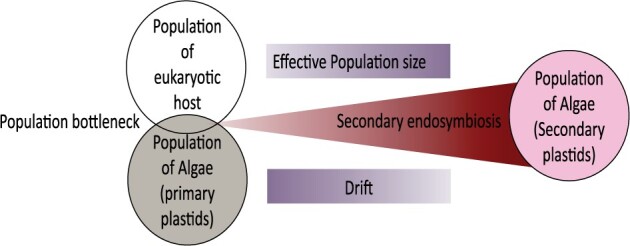
A general model for molecular dynamics during secondary green-type plastid endosymbiosis. The model illustrates the population bottleneck due to involvement of very small fraction of the actual population of the engulfed primary alga in secondary endosymbiosis. As the endosymbiont–host relationship ages, the effective population size increases, which counteracts the impact of stochastic drift, leading to establishment of secondary plastids after endosymbiosis.

Our results suggest a general model for the molecular dynamics of secondary plastid endosymbiosis (fig. 4). It is likely that a very small fraction of the actual population of the engulfed primary alga is involved in secondary endosymbiosis, creating a drastic population size bottleneck. This decrease in effective population size would then allow higher levels of drift to fix slightly deleterious mutations, a potential explanation for the long branches in the phylogeny of green plastids where secondary endosymbiosis events take place (Jackson et al. 2018).

Maintenance of the plastid genome during secondary endosymbiosis depends largely on nuclear-encoded proteins for DNA replication and repair (Smith and Keeling 2015). During secondary endosymbiosis, nuclear-encoded proteins are often transferred from the algal nucleus to the new host nucleus, with the product directed to the new plastid (Lane and Archibald 2008; Sarai et al. 2020). This might contribute to a period of reduced fidelity of plastid DNA replication during secondary endosymbiosis, which might in some cases lead to failure of the secondary plastid endosymbiosis. As the endosymbiont-host relationship ages, the drift acting on plastid genomes could eventually decrease, with higher effective population size and level of integration of plastid and host nucleus. This is reflected in the increased levels of selection on secondary plastids following endosymbiosis, emphasizing the important interplay between drift and selection during secondary endosymbiosis and their resulting impact on secondary plastid genomes.

### Three Independent Events

Our analyses comparing selection regimes of the three endosymbiosis events to the background individually showed distinctive scenarios. *Lepidodinium* showed the strongest relaxation (*k* = 0.3) followed by chlorarachniophytes (*k* = 0.45), indicating evidence of strongly relaxed selection during these two endosymbiotic events. However, euglenophytes showed a much lower level of relaxation (*k* = 0.86) during this endosymbiosis event.

Tightly constrained genome reduction along with evident purifying selection across all three green algal secondary endosymbioses emphasizes the evolutionary parallels among these independent events, but also clearly distinguishes the origin of secondary green plastids from other recently established obligate endosymbionts. Differences in degrees of relaxation and gene losses during these three secondary endosymbiosis events highlight different evolutionary paths. The host’s mode of nutrition may contribute to different selection pressures during plastid endosymbiosis. It is likely that if a mixotrophic lineage relies mostly on nutrients from phototrophy rather than phagotrophy, there might be stronger selection pressure on the plastid genomes of these lineages relative to other mixotrophic lineages that are not as reliant on phototrophy. Assessing the levels of mixotrophy for these lineages may contribute to explaining the underlying selection pressures.

It is also conceivable that differences in taxon sampling between euglenophytes (12 species), chlorarachniophytes (5 species), and *Lepidodinium* (1 species) could have influenced the results. In particular, if these groups contain currently undiscovered early-branching taxa, their inclusion might quantitatively alter the inferred rates of evolution and selection regimes (Heath et al. 2008). However, based on our current knowledge of biodiversity in the three groups of secondary plastids containing algae, most of the deeper branches have been sampled, lending credibility to our evolutionary inferences (Heath et al. 2008; Duchene et al. 2015). What we present here is thus the best achievable understanding from currently available data. Future discoveries of early-branching euglenophytes, chlorarachniophytes, green plastid dinoflagellates, and their close relatives might improve the precision of inferences, but it seems unlikely that this would result in radically different conclusions.

Because our analyses support increased drift, the differing relaxation intensity between the events implies that there may be differences in the impact of population bottlenecks underlying these events. Among the three events, euglenophytes are noticeable because they had the least relaxation of selection. Euglenophytes are distinct in many ways, including the possession of intron-encoded maturases (*mat1/ycf13*, *mat2*, and *mat5*) (Bennett and Triemer 2015; Dabbagh and Preisfeld 2017), and a plastid surrounded by three membranes. Chlorarachniophyte and *Lepidodinium* plastids have four membranes, with the innermost two membranes predicted to come from primary plastids and the outermost membranes derived from the current host. The absence of homologs of plastid-derived protein import components related to the outer membrane of primary plastids (TOC) hints at the possibility of the middle plastid membrane originated from the host in euglenophytes, and has led to speculation that integration of their plastids involved a novel/simplified process including proteins of host origin (Zahonova et al. 2018; Novák Vanclová et al. 2020). This could have facilitated more efficient integration of their plastid genomes, allowing faster recovery from a bottleneck. This may have enabled euglenophyte plastids to be integrated with less relaxation of selection.

## Materials and Methods

### Data Set

We used a phylogeny from a previous study, consisting of 151 green plastid genomes spanning the primary plastids of green algae (Chlorophyta, 133 genomes) and the secondary plastids of Euglenophyta (12 genomes), Chlorarachniophyta (5 genomes), and the dinoflagellate genus *Lepidodinium* (1 genome) (Jackson et al. 2018). After excluding primary lineages with incomplete or nonphotosynthetic plastids, we were left with 104 primary plastid lineages and all secondary plastid lineages for use in our gene loss analyses. Although secondary plastids of red ancestry are found in wider diversity than secondary green plastids, uncertainties surrounding the origin(s) and evolution of red algal complex plastids (Lane and Archibald 2008; Archibald 2009; Keeling 2013) would severely limit their utility for our goals, hence they were not considered. Our data set includes close extant relatives of ancestral green algae (plastid donors) that were involved in the secondary endosymbiosis events and an evolutionary timescale is available (Jackson et al. 2018), making this green plastid data set an outstanding case study to examine the molecular evolutionary dynamics associated with secondary endosymbiosis, and investigating differences and similarities among the three independent cases of secondary green plastid origination. Although this data set includes all the available secondary green plastids containing lineages at the time of construction, taxon sampling is somewhat biased towards euglenophytes.

### GC Content and Codon Bias

Basic features of the plastid genomes such as a number of coding sequences (CDS) and genome size were recorded and GC content of CDS and codon usage bias were calculated using the CodonO (Wan et al. 2007) function from the cubfits v.0.1-3 (Chen 2014) package in R v.3.5.1 (R Core Team 2018).

### Analysis of Gene Loss

Because we are working with genomes coming from different sources, we applied a strategy to obtain homologous protein sequences from the relevant taxa and excluded pseudogenes. Rather than relying on the gene names provided in the heterogeneous annotations, we clustered predicted CDSs based on sequence similarity into homologous groups, defined as “orthogroups,” using OrthoFinder version 1.4.0 at default parameters (Emms and Kelly 2015). A presence/absence matrix of the 199 orthogroups that were present across multiple species was constructed. Using this matrix and the reference phylogeny from Jackson et al. (2018), gene gain and loss along the phylogeny was estimated using PHYLIP version 3.695 (Felsenstein 2005), with the Dollo parsimony method and printing the states at all nodes of the tree. In a few cases, where single named genes were spread across two or more orthogroups, the orthogroups were merged. Gene loss along each branch was extracted from the PHYLIP output using OrthoMCL Tools (DOI 10.5281/zenodo.51349). The rate of gene loss per million years was calculated for each branch using the evolutionary time from the chronogram presented by Jackson et al. (2018). The estimated numbers of genes lost (and rates of gene loss) were ranked from largest to smallest to see if endosymbiotic branches had greater values compared with the background, and evaluated formally using ANOVA and Tukey HSD tests in the stats v3.6.2 package of R Core Team (2013). Of the 199 orthogroups, only 110 OGs corresponding to named genes with known function were conserved across most plastids, whereas the remaining OGs (mostly hypothetical genes of unknown function) were not examined further. To investigate if the genes lost during the secondary endosymbiosis may have been transferred to host nuclear genomes, we performed local tBLASTn searches using orthologous genes as query against the published nuclear genomes of *B.natans* (Curtis 2012) and *E.gracilis* (https://www.ncbi.nlm.nih.gov/assembly/GCA_900893395.1, last accessed October 11, 2021) (e-value cut-off = 1e−05).

### Selection Intensity Analysis

To study the variation in selection intensity in the protein-coding genes of secondary and primary green plastids, we used the hypothesis-testing framework RELAX (Wertheim et al. 2015) from the HyPhy software package version 2.3.14 (Kosakovsky Pond et al. 2005; Delport et al. 2010). This framework requires a predefined tree with subsets of test and reference branches specified. The subset of branches that are not set as test or reference remains unclassified. RELAX applies a branch-site model to estimate the strength of natural selection based on the ratio of nonsynonymous to synonymous substitutions (omega, *ω*) for three different *ω* categories (*ω*_1_ < *ω*_2_ ≤ 1< *ω*_3_) in the test and reference subsets. *ω* < 1 represents sites under purifying selection, *ω*  >  1 represents sites under positive selection and *ω* = 1 represents sites under neutral evolution. The relative selection intensity parameter (*k*) reflects intensification or relaxation of selection based on the relative proximity of *ω* values to 1 (neutral evolution). If *ω* values of test branches are closer to 1 than reference branches, then selection is relaxed (*k* < 1) and in the opposite scenario, selection has intensified (*k* > 1). The null model assumes identical *ω* values (*k* = 1) between test and reference branches. The alternative model fits different sets of *ω* values for test and reference, and thus *k* differs from 1, allowing a formal test of relaxed (*k* < 1) or intensified (*k* > 1) selection. The likelihood ratio test (LR) performed with *P*-value < 0.05 by comparing the null and alternate model quantifies statistical confidence for the obtained *k*-value.

### Models for Selection Analysis

We used the HyPhy-RELAX method to study molecular evolution through the process of endosymbiosis by designing different evolutionary models that allowed us to study aspects of selection intensity before, during and after the endosymbiosis process. For the selection analyses, we included only genes that were present in all of the lineages with secondary plastids (34 orthologous genes). The phylogenetic tree of algal green plastid genomes from Jackson et al. (2018) was used as the predefined tree on which test and reference branches were marked. In the phylogeny (fig. 1), branches leading to and connecting the species containing primary plastids (i.e., the green algae) were indicated as primary branches (P), and denote the state before secondary endosymbiosis. Secondary branches (S) are the branches leading to and connecting the species containing secondary plastids, and denote the state after secondary endosymbiosis. The endosymbiotic branches (E) indicate branches connecting the backbone of green algal lineages to the lineages with secondary green plastids, in other words, the branches along which secondary endosymbiosis took place (maroon-colored branches in fig. 1). Molecular evolution along the endosymbiotic branches includes the changes that took place during the transition of a permanently retained endosymbiont to a fixed organelle. Molecular evolution along the primary and secondary branches represents processes happening in primary plastids and established secondary plastids codiversifying with their new hosts, respectively. The *Lepidodinium* lineage includes only one plastid genome so we consider this branch as the endosymbiotic branch for this case.

Our first model, denoted “E × P + S,” has endosymbiotic (E) branches as the test set and all nonendosymbiotic branches (P + S) as the reference set. This model allows us to compare the selection intensity during endosymbiosis relative to before and after endosymbiosis. Our second model, denoted “S × P(E),” allowed us to evaluate differences in selection intensity between secondary (S) and primary (P) plastids, excluding the endosymbiont branches (E).

To study differences between individual endosymbiosis events, we fitted *E* × P + S models, but specifying only a single endosymbiotic branch as test (excluding all other endosymbiotic branches) and all nonendosymbiotic branches (P + S) as the reference set.

### Purifying Selection Analysis

Because functional plastid genes are expected to experience purifying selection, we also carried out an analysis to identify and quantify levels of purifying selection. The BUSTEC method implemented in HyPhy tests for alignment-wide evidence of conservation by fitting a random effects branch-site model to the entire phylogeny or a subset of tree branches (Murrell et al. 2015). The null model constrains *ω* values to greater than or equal to 1, excluding the possibility of purifying selection. The unconstrained model allowing *ω* values greater than and less than 1 serves as the alternate model. With endosymbiotic branches as the test branches, we used BUSTEC to fit the alternative unconstrained and null constrained models to these branches to quantify evidence for purifying selection during endosymbiosis.
